# Rest a while and run a mile: Relationship between distraction and negative emotions among college students in China

**DOI:** 10.1371/journal.pone.0236030

**Published:** 2020-09-11

**Authors:** Shi-Min Chen, Jie Fang, Li-Ming Wang, Cai-Li Liu

**Affiliations:** 1 School of Public Administration, China University of Mining and Technology, Xuzhou, China; 2 School of Humanities and Communication, Guangdong University of Finance & Economics, Guangzhou, China; 3 School of Foreign Language, China University of Mining and Technology, Xuzhou, China; 4 Psychological Counseling Center, Middle School Affiliated to China University of Mining and Technology, Xuzhou, China; University of Wuerzburg, GERMANY

## Abstract

Previous experimental studies have regarded distraction, an emotional regulation strategy, as an attentional disengagement strategy and considered it to be maladaptive in the long term. This study intends to further examine the relationship between distraction and negative emotions by using a questionnaire and a multiple mediation model. A total of 723 college students completed the distraction, cognitive reappraisal and problem-solving subscales of the Measurement of Affect Regulation Styles, the Needs Satisfaction Questionnaire, the Meaningful Life Measure, and the Emotional Experience Questionnaire of Well-being. Structural equation modeling (SEM) was performed, and mediation effects were tested. The results showed that (1) distraction was used significantly more frequently than problem-solving and cognitive reappraisal, with a large effect size (partial η^2^ = 0.321 > 0.138), and (2) distraction had an effect on negative emotions through two multiple mediation paths, i.e., positive emotion—cognitive reappraisal—meaning in life, and positive emotion—problem-solving—needs satisfaction. Distraction reduces negative emotions by enhancing positive emotions and facilitating cognitive reappraisal, problem-solving, meaning in life and needs satisfaction. It is not a kind of avoidance but a temporary rest to strive for a better life.

## Introduction

In recent years, the mental health of college students has become a social issue that has attracted an increasing amount of attention, especially with suicides among college students being reported from time to time in China. College students are in the developmental stage of emerging adulthood, which is situated between adolescence and adulthood, beginning at age 18 and continuing through the mid-twenties [1]. They should not only focus on their academic courses but also be engaged in identity exploration, particularly in the areas of love and work [2]. They are also confronted with the great pressure of searching for jobs or taking postgraduate entrance exams before graduation. All these factors lead to many negative emotions, such as anxiety and depression, which need to be mitigated or eliminated. This study aims to explore the effect of distraction, an emotional regulation strategy, on negative emotions.

### Stressors and negative emotions of college students

At present, college students are under a variety of pressures in China and experience all kinds of negative emotions. The first stressor is academic distress. The courses are difficult, and academic stress is the top source of concern for science and engineering and medical students [3–5]. In addition, many college students face the great pressure of fierce academic competition [6]. The enormous academic pressure often makes college students anxious.

The second stressor is financial stress. The expenses of studying and living are high for college students. Moreover, as college students come from families with different socioeconomic statuses, social comparison is common. Impoverished college students experience significantly higher levels of anxiety, self-inferiority and depression than those of non-poor college students [7]. A survey showed that 23.6% of poor college students in China have depressive symptoms [8].

The third pressure comes from disharmonious families. There are a large number of left-behind families in China. The self-esteem of the college students with left-behind experience is significantly lower than that of those college students without non-left-behind experience, and the levels of anxiety and depression are significantly higher among left-behind college students [9, 10]. In addition, the divorce rate is increasing annually in China. Parents' divorce has substantial negative effects on the mental health of their children. College students from divorced families report significantly higher levels of interpersonal sensitivity, anxiety, depression and hostility than those from normal families, especially when the parents divorced when the child was aged between 4 and 7 years old [11].

Fourth, as an increasing number of college students fall in love, two problems often arise. The first is dating violence, which refers to physical, sexual, or psychological/emotional violence which is stalking or occurring between current or former teen dating partners [12]. College students who experienced abuse in childhood reported more dating violence than those who did not [13]. The relationship between dating violence and mental health varies by direction (perpetration vs. victimization) and type of violence. Victimization is associated with anxiety, depressive symptoms, and hostility, while perpetration is associated with hostility [14]. Experiencing multiple acts of violence may lead to more severe depressive and anxiety symptoms than experiencing psychological, physical, or sexual abuse in isolation [14]. The second problem is premarital sexual behavior. Female college students may have the following negative emotions after premarital sexual behavior: (1) shame, regret, disgust and other negative sexual experiences; (2) worry about the behavior being revealed to others, which results in negative social evaluation; (3) worry about pregnancy and sexually transmitted diseases; and (4) worry about harming their future marriage [15]. And what’s worse is unplanned pregnancies that greatly do harm to the physical and mental health of female college students and cause anxiety and depression among them [16].

Last but not least, for many reasons, college students are under great pressure to search for jobs [17]. The first reason is that there are a huge number of college graduates every year. Taking 2019 as an example, the number of university graduates in China was 8.3 million. The second is that there is a certain gap between the major settings, cultivation mode in school and the demand in society. The third is that family members often have high expectations for college students. All these factors elicit negative emotions such as anxiety, confusion, self-inferiority, and a sense of frustration among college students [18].

In short, Chinese college students suffer from various stressful events, including academic distress, financial stress, disharmonious families, dating violence, premarital sexual behavior, and job-hunting pressure, which lead to a variety of negative emotions, such as anxiety, depression, self-inferiority, hostility, interpersonal sensitivity, shame, confusion, worry, and frustration.

### The concept of distraction

A variety of strategies are adopted to address the negative emotions of college students. These strategies include distraction, cognitive reappraisal, emotional expression, problem-solving, and avoidance [19]. Previous studies have mainly focused on the effect of cognitive reappraisal, emotional expression, and problem solving on negative emotions [20–23]. However, few studies have examined distraction. We would like to further explore the effect of distraction on negative emotions in this study.

Compas, Connor-Smith, Saltzman, Thomsen, and Wadsworth (2001) classified coping strategies into engagement and disengagement coping [24]. Disengagement coping refers to responses that are oriented away from the stressor or one's emotions or thoughts [24]. Disengagement is not purely avoidance, with which the respondents abandon efforts and redirect their attention toward an alternative target. Disengagement also includes distraction, with which the respondents just take their attention away from the stressful events temporarily, but still maintain awareness and acknowledgment of the stressors. People often distract their attention by engaging in various activities that are interesting and relaxing in daily life. Therefore, Larsen and Prizmic (2004) defined distraction as the act of disengaging from a problematic situation or avoiding thinking about a problem and instead engaging in somewhat low-effort but preoccupying activities (e.g., watching television, listening to music, working on a hobby, or reading books) in an effort to get one’s mind off a negative event or emotion [19].

### Distraction and negative emotions

Two types of methods have been used to study the relationship between distraction and negative emotions: experimental method and questionnaire method. Experiments have shown that distraction requires only a small amount of cognitive resources for emotion modulation and can decrease negative emotion quickly, especially in high-intensity negative situations [25–27]. Distraction is considered to be an avoidance strategy [25] or an attentional disengagement strategy [26], which is adaptive in the short term for both high and low emotional intensities but can be maladaptive in the long term. In previous experiments, distraction has been induced by assigning arithmetic problems to participants to disengage them from the negative emotions [25] or thinking about something that is emotionally neutral [26, 27]. However, it is well known that few people would distract themselves from negative emotions in these ways when they are in bad mood in daily life. Most of us would like to distract ourselves by taking part in some activities that we are interested in. Therefore, the conclusion drawn by experimental studies that distraction is maladaptive in the long term is problematic.

The questionnaire method assesses distraction in a way that is closer to daily life than the experimental method. Pottie (2008) conducted an investigation of 93 parents of children with autism and found that the frequency of distraction significantly negatively predicted negative emotions [28]. However, only regression analysis was used in Pottie’s study. The mechanism of how distraction alleviates negative emotions is not clear. We would like to further explore the relationship between distraction and negative emotions by using a multiple mediation model in this study, in which the independent variable (X) affects a dependent variable (Y) through multiple potential intervening variables or mediators (M) [29].

### Hypotheses

#### The mediator of positive emotions

When individuals devote themselves to somewhat low-effort but preoccupying activities, they can not only decrease negative emotions directly by disengaging from the stressful situation or avoiding thinking about the problem, but also increase various types of positive emotions. Previous studies have shown that all kinds of entertainment activities are beneficial to produce a variety of positive emotions. Music that is in a major mode induces an emotional response similar to a pleasant experience or happiness [30]. Dancing brings many positive feelings, such as joy, delight, euphoria, and self-confidence, to people and promotes social interaction with others, which facilitates a sense of belonging [31]. In addition, recreational sport activities [32] and nature-based outdoor recreation, such as walking in green and natural environments [33], also produce many positive emotions, such as relaxation and aesthetic feelings. The broaden-and-build theory of positive emotions suggests that positive emotions can undo the lingering effect of negative emotions [34, 35], and the Evaluative Space Model proposes that positive emotions can uncouple negative emotions [36, 37]. Therefore, we propose the following simple mediation hypothesis:

H1: Positive emotions mediate the effect of distraction on negative emotions.

#### The mediator of cognitive reappraisal and meaning in life

According to the broaden-and-build theory of positive emotions, positive emotions can broaden people's momentary thought repertoires and widen the array of thoughts [34, 35]. Positive emotions resulting from distraction can facilitate individuals to reinterpret stressful events. When confronted with stressful situations, people can create a more intentional and purposeful way of living through re-evaluating and focusing on what is truly important, connecting with the transcendent, or developing a heightened sense of appreciation for life [38]. Such an adaptive type of coping may lead individuals to affirm or develop new sources of meaning in their lives, leading them to perceive their lives as more meaningful [38]. On the other hand, those who cope less effectively or employ generally maladaptive strategies such as avoidance or denial may miss the opportunities to develop wisdom or insight through their struggle and may therefore subsequently report lower levels of meaning in life [38].

Victor Frankl (1963) proposed that individuals are strongly motivated to find personal meaning, that is, to understand the nature of their lives; to feel that life is significant, important, worthwhile, or purposeful; and to avoid a pathological condition characterized by apathy, boredom, and aimlessness [39]. Meaning in life refers to a multidimensional construct consisting of the cognition of order, coherence, and purpose in one’s existence; the pursuit and attainment of worthwhile goals; and the accompanying sense of fulfillment [40]. Previous studies have shown that meaning in life is a protective factor against mental illness and distress [41]. Those with meaning in life feel more hopeful and less depressed and therefore are willing to struggle for a better life [42, 43]. We hereby present the following multiple mediation hypothesis:

H2: Cognitive reappraisal and meaning in life mediate the effect of positive emotions on negative emotions.

#### The mediator of problem-solving and need satisfaction

Moderate-intensity positive emotions can broaden cognitive scope and facilitate creative problem-solving [44, 45]. Neuropsychological studies have found that positive emotions can augment the level of dopamine in the ventral tegmental area (VTA) [46, 47]. Projections from the VTA to the prefrontal cortex and the anterior cingulate are important because they provide a direct mechanism through which positive affect can influence cognition. Evidence suggests that dopamine projection to the prefrontal cortex facilitates working memory whereas dopamine projection to the anterior cingulate facilitates executive attention and cognitive perspective selection, enabling cognitive flexibility and problem-solving. In other words, positive emotions can help individuals to think out more ways to deal with the stressful circumstances.

When stressful events are solved, individuals’ lives can be improved by satisfying quite a few needs. When college students are faced with academic distress, they make substantial efforts to enhance their academic performance by consulting teachers and classmates frequently and improving their learning methods, which can help them not only master knowledge and skills but also win appreciation and respect from teachers and classmates, thus fulfilling the needs of competence and respect. When college students are under financial stress, they get part-time jobs or scholarships by studying hard to support themselves, which is beneficial for fulfilling the needs of competence, respect, physiology and so on. When college students suffer from interpersonal disturbances, they learn the rules of interpersonal communication and improve their interpersonal skills, which is helpful for fulfilling the needs of competence and affiliation. When college students are faced with job-hunting pressure, they find jobs by analyzing their own advantages and disadvantages, making elaborate resumes and actively searching for employment information, thus greatly improving their self-confidence and social status and meeting the needs of respect. When various needs are met, negative emotions such as anxiety, depression, and anger can be greatly reduced [48]. Therefore, we posit the following multiple mediation hypothesis:

H3: Problem-solving and need satisfaction mediate the effect of positive emotions on negative emotions.

**Fig 1:**
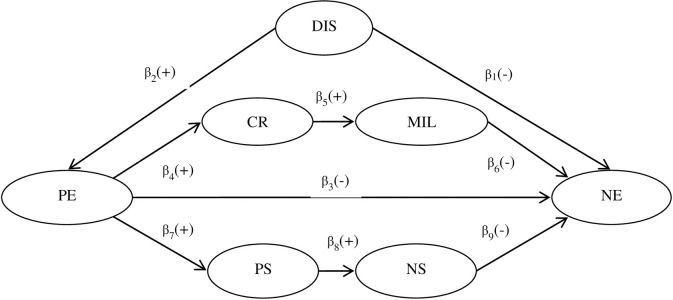
Hypothetical multiple mediators model between distraction and negative emotions. DIS = distraction; PE = positive emotions; CR = cognitive reappraisal; MIL = meaning in life; PS = problem-solving; NS = needs satisfaction; NE = negative emotions.

Based on hypotheses 1, 2 and 3, we propose the following hypothetical multiple mediation model (H4) shown in Fig 1, which encompasses three mediation effects: (1) simple mediation through the mediator of positive emotions; (2) multiple mediation through the mediators of positive emotion, cognitive reappraisal, and meaning in life; and (3) multiple mediation through the mediators of positive emotion, problem-solving, and needs satisfaction.

## Methods

### Procedure

The procedure included the following steps. First, we obtained ethical approval from the Research Ethics Committee of China university of Mining and Technology (NO.: 201904007). Second, the questionnaires were uploaded to the “Questionnaire Star” website, the most widely used questionnaire website in China, which produced a two-dimensional code of the questionnaire. The code was presented on a presentation slide of the software PowerPoint. Third, we connected college teachers in six colleges, and sent the presentation slide with the two-dimensional code of the questionnaire to them. Fourth, the survey was made in classes. Students were told that their participation in the study was voluntary, that they were free to withdraw at any time and that their responses would be kept confidential. They were asked to provide written informed consent after the procedures had been fully explained. Finally, the students scanned the two-dimensional code of the questionnaire with the APP Wechat in their mobile phone, and began to fill in the questionnaire. After completing the questionnaire, students were given a little gift.

### Participants

Questionnaires were deemed invalid and were eliminated in cases of inconsistent responses to lie-detection items (“Facing my daily task is a painful and boring experience” and “my daily living is dull and routine”). A total of 723 valid questionnaires were obtained. The college students consisted of 311 male students and 412 female students and comprised 312 liberal arts students and 411 science and engineering students. The average age of the sample was 20.12 years (SD = 1.37), which contained 280 freshmen, 135 sophomores, 144 juniors and 164 seniors.

### Sample size

The sample size for a study is very important in any research because it influences many aspects of a study, including the suitability of methods for use (e.g., parametric/nonparametric methods), model fit, and the precision and power of the model’s parameter estimates. Strategies for determining sample size have been discussed in previous studies. One rule of thumb is that a sample size of fewer than 100 is often considered small, a sample size between 100 and 200 is medium, and a sample size exceeding 200 is large; this notion is referred to as the N ≥ 100 rule. Another approach is to consider model complexity in terms of the ratio of sample size to the number of free parameters needed to be estimated in a model. A minimum sample size would be at least 10 times the number of free model parameters; this notion is referred to as the N: q ≥ 10 rule. However, these approaches are not dependable because the requisite sample size is a function of numerous factors, including the amount and patterns of missing data, the strength of the relationships among the indicators, the types of indicators (e.g., categorical or continuous), the estimation methods (e.g., [robust] maximum likelihood, robust weighted least squares), and the reliability of the indicators. Instead of elaborating general guidelines for sample size, more empirically grounded, individual-model-focused approaches to determining sample size in relation to parameter precision and power have been proposed. Muthén and Muthén (2002) argued for the use of Monte Carlo analysis [49], where sample size is estimated under various conditions by taking into account the statistical precision and power of individual parameter estimates and various data conditions. They argue that the Monte Carlo method is flexible in that they can test with pinpoint accuracy the precision and power of individual parameter estimates in a model by taking into account non-normality, the type of indicators (i.e., binary, categorical, and continuous), and the amount and patterns of missing data.

There are five criteria to determine if the sample size for a model is sufficient in terms of precision and power [49, 50]. First, parameter bias should not exceed |10%| for any parameter in the model. Parameter bias is equal to the difference between the population parameter and the average sample parameter multiplied by 100%. Second, standard error bias should not exceed |10%| for any parameter in the model. The standard error bias is equal to the difference between the standard deviation of the sample parameters and the standard error of the sample parameters. Third, the standard error bias for the parameter for which power is of particular interest should not exceed |5%|. Monte Carlo simulation using Mplus presents the results of the mean square error of the parameters. Fourth, 95% coverage—the proportion of replications for which the 95% confidence interval covers the population parameter value—should fall between 0.91 and 0.98. Fifth, power exceeds 0.80—a commonly accepted value for sufficient power. Monte Carlo simulation using Mplus provides the results of the percentage of replications for which the parameter is significantly different from zero (i.e., the power estimate of a parameter).

The number of observations was set as 723, and Monte Carlo analysis was performed using Mplus 7.0 in this study. The maximum parameter bias was 2.1%, and the maximum standard error was 5.6%, both of which were less than 10%. The maximum mean square error of the parameters was 0.38%, which was less than 5%. The least 95% coverage was 0.942, which was greater than 0.91. The lowest power estimate of the parameters was 0.822, which was greater than 0.8. These results showed that the sample size of 723 was sufficient in terms of precision and power for this study.

### Measurement of positive emotions

Positive emotions were assessed in this study not by measuring the strength of positive emotions directly produced by distraction but by measuring the frequency of positive emotions experienced in daily life. When the questionnaire method is used, it is impossible to measure the positive emotions following each instance of distraction. Thus, we assess general positive emotions in daily life, which is not sufficiently accurate from the perspective of studying causal relationships. However, it can conveniently provide an approximate relationship between distraction and positive emotions among people in daily life with a large sample size. This method has been employed in previous studies [28, 51].

### Measures

#### Distraction, cognitive reappraisal and problem solving

Distraction, cognitive reappraisal and problem solving were assessed with the 10-item distraction subscale, 8-item cognitive reappraisal subscale and the 3-item problem-solving subscale from the Measurement of Affect Regulation Styles [19]. Example items include the following: “I watch TV, read a book, etc., for distraction”, “I try to reinterpret the situation to find a different meaning”, “I take action to solve the problem causing my mood”. Responses were made on a 5-point scale ranging from 1 (never) to 5 (always), indicating the frequency that the participants used the following strategies in the face of stressful events over the course of the past year. The English version was translated into Chinese by three psychology professors and doctors and one professional translator and was then backtranslated by another professional translator. This translation and backtranslation process was repeated before the final form was established. The results of confirmatory factor analysis were as follows: χ^2^/df = 3.68 < 8.0, CFI = 0.941 > 0.90, TLI = 0.931 > 0.90, and RMSEA = 0.048 < 0.08, which showed that the fit of all indices met the cut-off criteria. The reliabilities of the three subscales in this study were Cronbach α = 0.871, 0.850, and 0.825; all of which were greater than 0.80, indicating good reliability.

#### Needs satisfaction

Needs satisfaction was assessed with a 14-item Needs Satisfaction Questionnaire [48]. The Chinese revision of the Needs Satisfaction Questionnaire was revised by Chen, Gao, Zhang and Sun (2017) [52]. This questionnaire measured six kinds of needs satisfaction, including the satisfaction of needs for physiology (e.g., “I have enough money for shelter“), safety (e.g., “I feel safe walking alone”), love and belonging (e.g., “I have others that I can count on for help in an emergency“), respect (e.g.,”I am treated with respect by others”), autonomy (e.g.,”I can make choices based on my wishes”), and competency (e.g., “I can manage the many responsibilities of my daily life“) needs. Responses are made on a scale ranging from 1 (strongly disagree) to 5 (strongly agree). The reliability of the scale in this study was Cronbach *α* = 0.830.

#### Meaning in life

Meaning in life was measured with a 23-item Meaningful Life Measure [53]. The Chinese revision of Meaningful Life Measure was revised by Chen, Gao, Zhang and Sun (2017) [52]. Five dimensions were used to measure meaning in life, that is, exciting life (e.g., “My life interests and excites me”), accomplished life (e.g., “I find it satisfying to think about what I have accomplished”), principled life (e.g., “The beliefs I hold about the world enable me to make sense of my existence”), purposeful life (e.g., “I have a clear idea of what my future goals and aims are”), and valued life (e.g., “My life is significant”). Responses were made on a scale ranging from 1 (strongly disagree) to 5 (strongly agree). The reliability of the questionnaire in this study was Cronbach α = 0.923.

#### Positive and negative emotions

Positive and negative emotions were assessed with a 27-item Emotional Experience of Well-being Questionnaire [54], which included fourteen positive emotions (e.g., “warmth” and “hope”) and thirteen negative emotions (e.g., “anxiety” and “depression”). Responses were made on a 5-point scale indicating the frequency in which the participants have experienced each emotion in daily life over the course of the past year, ranging from 1 (never) to 5 (always), and the reliability of the scale in this study was Cronbach α = 0.899.

### Statistical method

The product-of-coefficients and bias-corrected nonparametric percentile bootstrapping methods were used to test the multiple mediation effect in this study [29, 55]. Structural equation modeling (SEM) was conducted with the software Mplus 7.0 due to this method’s ability to account for measurement error and manage multiple endogenous constructs. The test procedure was as follows [55–57]. First, confirmatory factor analysis (CFA) for measurement models was performed to test whether the indicators load significantly onto the underlying factor. Second, structural equation modelling was constructed, and the values of fit were evaluated. The following goodness-of-fit indices were used [58]: absolute fit indices, including χ^2^/df and RMSEA, and comparative fit indices, including CFI and TLI. The cut-off criterion for χ^2^/df was 8 for an “acceptable” fit. The cut-off criterion for RMSEA was 0.08 for an “acceptable” fit. The cut-off criteria for CFI and TLI were both 0.90. Third, the bias-corrected nonparametric percentile bootstrap method was used to test the significance of the chain medication effect by drawing 1000 bootstrap samples with replacement from the full dataset [59]. These samples were then used to construct a 95% confidence interval for the indirect effects. If this interval did not contain zero, the indirect effect was significant at p < 0.05. Fourth, the mediation effect size was calculated. There is not an ideal index of the mediation effects of an inconsistent mediation model in which the indirect effect ab and the direct effect c’ have opposite signs [60]. The absolute value of ab/c’ (|ab/c’|) is recommended as the index of the mediation effects until a better index can be found.

## Results

### Descriptive statistics and bivariate correlations

The means, standard deviations, and correlations are shown in Table 1. Distraction was significantly positively associated with cognitive reappraisal, problem-solving, meaning in life, needs satisfaction and positive emotion and was negatively correlated with negative emotion. One-way within-subjects ANOVA showed that distraction was used significantly more frequently than problem-solving, which in turn was used significantly more frequently than cognitive reappraisal, with a large effect size (partial η^2^ = 0.321 > 0.138).

**Table 1:** Means, standard deviation, and bivariate correlation.

Variables	1	2	3	4	5	6	7
DI	1						
CR	.543**	1					
PS	.443**	.506**	1				
MIL	.395**	.389**	.631**	1			
NS	.427**	.307**	.426**	.549**	1		
PE	.400**	.380**	.399**	.566**	.558**	1	
NE	-.195**	-.148**	-.142**	-.395**	-.442**	-.377**	1
M	3.53	3.05	3.36	3.22	3.60	3.12	2.32
SD	0.80	0.73	0.74	0.62	0.48	0.77	0.68

n = 723

** *p* < 0.01

*** *p* < 0.001, DI = distraction; CR = cognitive reappraisal; MIL = meaning in life; PS = problem-solving; NS = need satisfaction; PE = positive emotion; NE = negative emotion.

### Multiple mediation test

Confirmatory factor analysis (CFA) showed that all of the fit indices of the measurement models met the cut-off criteria. Structural equation model 1, 2, and 3 were constructed according to H1, 2, and 3. Table 2 shows the model fit indices, the values of mediation effects (a_1_a_2_ for Model 1, and a_1_a_2_a_3_ for Model 2 and 3), the test results of mediation effect of Model 1, 2, and 3. It could be seen that the model fit indices all met the cut-off criteria (χ^2^/df < 8.0, CFI > 0.90, TLI > 0.90, RMSEA < 0.08), suggesting that the models were acceptable; the bootstrap confidence intervals of mediation effects were all below 0, suggesting that the mediation effects were significant. Therefore, H1, 2, 3 were all validated.

**Table 2:** The model fit indices and the test results of mediation effects of Model 1, 2, 3.

Model	χ^2^/df	CFI	TLI	RMSEA	a_1_a_2_(a_3_)	CI
Model 1	3.67	0.930	0.920	0.051	-0.201	[-0.276,-0.148]
Model 2	4.26	0.922	0.912	0.057	-0.152	[-0.230,-0.099]
Model 3	4.17	0.934	0.920	0.061	-0.132	[-0.191,-0.087]

**Fig 2:**
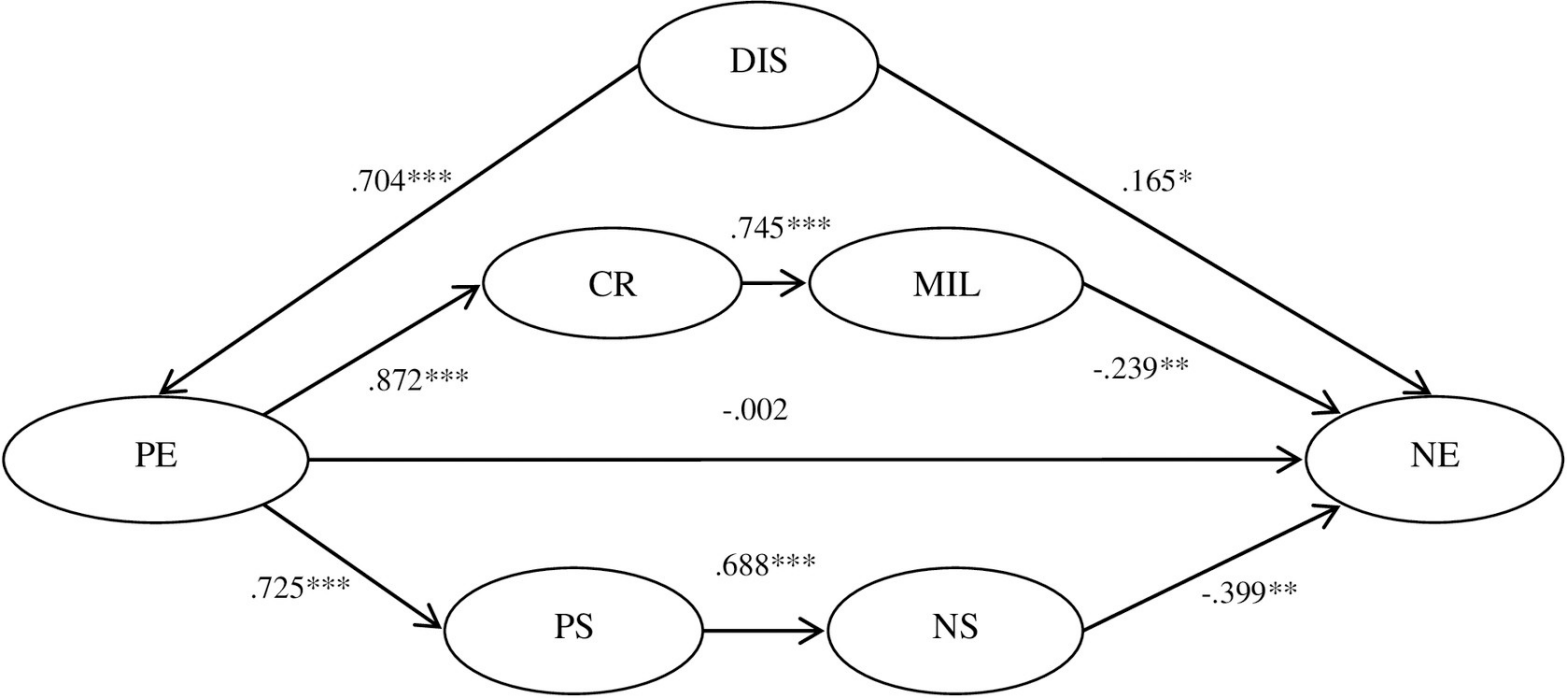
Multiple mediators model between distraction and negative emotions. * < 0.05, ** *p* < 0.01, *** *p* < 0.001, DIS = distraction; PE = positive emotions; CR = cognitive reappraisal; MIL = meaning in life; PS = problem-solving; NS = needs satisfaction; NE = negative emotions.

Next, structural equation model 4 was constructed according to H4. The model fit indices were as follows: χ^2^/df = 4.55 < 8.0, CFI = 0.913 > 0.90, TLI = 0.904 > 0.90, and RMSEA = 0.064 < 0.08, which all met the cut-off criteria, suggesting that the model was acceptable. The standard path coefficients are indicated in Fig 2.

The bias-corrected nonparametric percentile bootstrap method was used with 1000 times of resampling, and 95% confidence intervals were estimated. The confidence intervals, mediation effects, and mediation effect sizes are shown in Table 3. The confidence intervals of the direct effect and the mediation effect through the mediators of positive emotion contained 0, indicating that these two effects were not significant. The confidence intervals of the chain mediation effect through the mediators of positive emotions, cognitive reappraisal and meaning in life did not contain 0, and so did the confidence intervals of the chain mediation effect through the mediators of positive emotions, problem-solving and need satisfaction, indicating that these two chain mediation effects were significant. The confidence interval of the total indirect effect also did not contain 0, showing that the total indirect effect was significant. The mediation effect sizes of the two chain mediation effects and total indirect effect were 1.16, 1.49, and 2.67, respectively.

**Table 3:** Test results of the mediation effect of model 4.

Effects	Mediators	95% CI	Med_effect	Med_effect size
Direct effect		[-0.088, 0.277]	0.094	
Med_effect	PE	[-0.191, 0.189]	-0.001	0.01
	PE-CR-MIL	[-0.197, -0.022]	-0.109	1.16
	PE-PS-NS	[-0.237, -0.059]	-0.140	1.49
	Total med_effect	[-0.374, -0.127]	-0.251	2.67
Total effect(c)		[-0.282,-0.031]	-0.156	

Med_effect = mediation effect; PE = positive emotions; CR = cognitive reappraisal; MIL = meaning in life; PS = problem-solving; NS = needs satisfaction

## Discussion

### Effect of distraction on negative emotions

This study distinguished two different disengagement strategies (avoidance and distraction). Distraction is the act of "getting some distance" from current duties or demands. It allows mental relaxation from current strain, thereby helping individuals to recharge internal batteries and thus enabling them to refocus on the task at hand. The aim of this study was to explore how distraction affects negative emotions. We first compared the frequency of the use of distraction, cognitive reappraisal and problem-solving and explored their correlations, and then, we used a multiple mediation model to examine the potential mediators of the effect of distraction on negative emotions, including positive emotions, cognitive reappraisal, meaning in life, problem-solving, and needs satisfaction.

Tables 1 and 4 show that the strategy of distraction was used more frequently than problem-solving and cognitive reappraisal. A possible explanation for this finding is that distraction requires only a few cognitive resources for modulation and can decrease negative emotions quickly; thus, people tend to use distraction most frequently, whereas cognitive reappraisal requires a maximum of cognitive resources for modulation and is used minimally. The correlational analysis showed that distraction was significantly associated with cognitive reappraisal and problem-solving (Table 1), which indicated that the three coping strategies were not independent but related to each other to some extent.

**Table 4:** Comparison among distraction, cognitive reappraisal, and problem-solving.

DIS(1)		CR(2)		PS(3)		F	Post Hoc	Partial η^2^
M	SD	M	SD	M	SD			
3.53	0.80	3.05	0.73	3.36	0.74	150.7***	1>3>2	0.321

DIS = distraction = 1; CR = cognitive reappraisal = 2; PS = problem-solving = 3

*** *p* < 0.001; > refers to “significantly greater than”

Table 1 indicates that distraction was significantly associated with the frequency of negative emotion, which was similar to Pottie’s study [28]. The multiple mediation analysis indicated that distraction influenced negative emotions through the following four pathways. The first pathway was the direct path from distraction to negative emotions. Contrary to our hypothesis, the path coefficient was positive, although the direct effect was not significant (β = 0.094, CI [-0.088, 0.277]), which might be explained by the fact that distraction may require time and money. For college students, the money they spend mainly comes from their parents, and some of them are faced with financial stress; thus, the costs of time and money directly produce pressure and negative emotions. The second pathway was through the mediator of positive emotions. The mediation effect was not significant (CI [-0.188, 0.064]) with the mediation effect size of -0.001, which showed that the effect of distraction on negative emotion was not through the role of positive emotion undoing or uncoupling negative emotions in the long run. The third pathway was through the mediators of positive emotion, cognitive reappraisal and meaning in life, and the fourth pathway was through the mediators of positive emotion, problem solving and needs satisfaction. Both multiple mediation effects were significant, with mediation effect sizes of 1.16 and 1.49, respectively, indicating that distraction had an important impact on negative emotions through these two paths. On the whole, distraction had a negative impact on negative emotions with a total effect size of -0.156, indicating that distraction effectively reduced negative emotions in daily life.

Previous experiments only explored the second pathway of the effect of distraction on negative emotions, which showed that distraction had no long-term effect on negative emotions directly through positive emotions. This study used a multiple mediation model to examine the four pathways through which distraction affected negative emotions, and pointed out that distraction had a long-term effect on negative emotions through the third and fourth pathways of the enhancement of positive emotions facilitating cognitive reappraisal, problem-solving, meaning in life and needs satisfaction.

### Practical implication

Just as the proverbs goes, “If you feel exhausted, you can take a rest, but not give up”, “Rest a while and run a mile”. Distraction is not a kind of avoidance but a temporary break to enhance one’s fight for life. When college students encounter negative life events and are subject to negative emotions, regular distraction helps elicit their positive emotions, which contributes to the change in their views on the events, and helps to solve the problems to improve their meaning in life, meet their needs, and finally alleviate their negative emotions. Therefore, it is advisable for college students to cultivate hobbies and form the habit of relaxation, which is helpful for effectively reducing negative emotions and coping with the pressure of daily life.

### Highlights

This study yielded the following highlights. First, this study distinguished two different disengagement strategies (avoidance and distraction). Previous studies often considered that all subtypes of disengagement strategies were maladaptive. This study argued that distraction is a temporary attentional disengagement resulting in mental relaxation from current strain, whereby the respondents prepare to refocus on the stressors more effectively, which is adaptive on many occasions. Second, previous experimental studies regarded distraction as an avoidance strategy or an attentional disengagement strategy and considered it to be maladaptive in the long term. This study employed a questionnaire method, which was close to daily life, and examined the effect of distraction on negative emotions through multiple mediators, including positive emotions, cognitive reappraisal, meaning in life, problem-solving, and needs satisfaction, which further revealed relationship between distraction and negative emotions. Third, distraction, cognitive reappraisal and problem-solving were regarded as independent emotional regulation strategies in previous studies. This study showed that different emotional regulation strategies interact with each other to some extent.

### Limitations and future directions

Despite its contributions, the limitations of this study should be acknowledged. The first limitation is that due to the questionnaire method, positive emotions were assessed in this study not by measuring the strength of positive emotions directly produced by distraction but by measuring the frequency of positive emotions experienced in daily life. The questionnaire method has the advantage of conforming to real life, but it was a correlational study in nature and cannot explain the causal relationship in a strict way. Future studies can utilize a longitudinal design to supplement this research, which may better explain the causal relationship. In contrast, experimental method can better provide a causal explanation, but the measurement of distraction in previous experiments was not in line with the real life. In the future, experimental methods may be improved to further explore the relationship between distraction and negative emotions.

In addition, it should be remembered that not all people would use cognitive reappraisal and problem-solving following distraction in the face of stressful events. If distraction is used excessively or exclusively, it may be maladaptive in the long term. Previous studies showed that those with high neuroticism easily suffer from cognitive failures, especially under stressful conditions [61, 62]. High-neuroticism individuals might employ less cognitive reappraisal and problem-solving following distraction because of cognitive failure in the face of stressful events. In future studies, data can be collected to verify this assumption.

## Conclusion

In summary, distraction was used more often than problem solving and cognitive reappraisal in daily life because distraction requires few cognitive resources for modulation and can decrease negative emotions in a short time. Distraction is not a kind of avoidance but a temporary rest to strive for a better life. Distraction reduces negative emotions by the mediating effect of enhancing positive emotions and facilitating cognitive reappraisal, problem-solving, meaning in life and needs satisfaction. When college students encounter negative life events and generate negative emotions, regular distraction helps them improve their positive emotions, change their views on the events, and find ways to solve their problems to better improve the meaning of their lives, meet their needs, and finally alleviate their negative emotions. Therefore, it is desirable for college students to cultivate hobbies and form the habit of relaxation, which is helpful for effectively reducing negative emotions and coping with the pressure of daily life.

## Supporting information

S1 Data The original data of this study with the SPSS files. (ZIP)
